# Effect of long-term pharmacological treatments on Alzheimer disease: A systematic review and network meta-analysis

**DOI:** 10.1097/MD.0000000000039753

**Published:** 2024-09-20

**Authors:** Xiaoyan Deng, Daishun Li

**Affiliations:** a Department of Rehabilitation Medicine, Chengdu Tianhui Community Health Service Center, Sichuan, China; b Department of Rehabilitation Medicine, Sichuan Provincial People’s Hospital, University of Electronic Science and Technology of China, Sichuan, China.

**Keywords:** Alzheimer disease, long-term, network meta-analysis, pharmacological

## Abstract

**Background:**

To analyze and compare the pharmacological treatments for Alzheimer disease (AD), we will conduct a systematic review and network meta-analysis focusing on their efficacy and safety over a duration exceeding 1 year.

**Methods:**

We searched the databases of PubMed, Scopus, EMBASE, Web of Science, the Cochrane Central Register of Controlled Trials, and CNKI until July 30, 2023, for randomized controlled trials (RCTs) evaluating pharmacological treatments for AD.

**Results:**

Seventeen RCTs, comprising 7214 participants, investigated the efficacy of the following drugs: Donepezil, Rivastigmine, Galantamine, Memantine, Ginkgo biloba extract (EGb), Atorvastatin-calcium and Vitamin B in the treatment of AD. The network meta-analysis resulted indicated that placebo demonstrated greater effectiveness compared to Atorvastatin-calcium 80 mg (mean different [MD] = ‐6.93, confidence interval [CI] −11.57, −2.29) and Rivastigmine 12 mg (MD = ‐3.33, CI −6.56, −0.09). EGb120 mg exhibited a greater improvement in cognition compared to Atorvastatin-calcium 80 mg (MD = 7.77, CI 2.07, 13.46) and Rivastigmine 12 mg + EGb120 mg (MD = 9.92, CI 1.32, 17.22). EGb 120 mg emerged as the most efficient intervention for cognition, while placebo demonstrated the least harm over a period exceeding 1 year.

**Conclusions:**

In this network meta-analysis of studies of patients with AD and a follow-up period of at least 1 year, EGb 120 mg demonstrated cognitive benefits, while placebo posed the least harm for AD. More RCTs are required to address the uncertainty surrounding the efficacy of medication.

## 1. Introduction

Alzheimer disease is a chronic, progressive, age-related neurodegenerative disease characterized by acquired cognitive decline, which progressively impairs activities of daily living, leading to dependence, disability and ultimately, death.^[1]^ According to the World Alzheimer’s Disease Report 2018, dementia develops in 1 person worldwide every 3 seconds. Currently, there are at least 50 million people worldwide living with dementia, a number projected to reach 131.5 million by 2050.^[2]^ Among them, approximately 60–70% are afflicted with Alzheimer disease (AD). As the disease progresses, individuals lose the capacity for self-management and require long-term care, imposing a significant burden on patients, their families, society, and the healthcare system.^[1]^ AD ultimately results in death, with a median survival period of only 7 years following diagnosis. Dementia typically manifests in individuals over the age of 60, ranging from 5 to 7%.^[3,4]^

AD currently have no cure. The current drugs for AD treatment include cholinesterase inhibitors (Donepezil, Galantamine, Rivastigmine) and the N-methyl-d-aspartate receptor antagonist, Memantine.^[5]^ Additionally, some adjuvant drugs Vitamins B are also utilized in treatment protocols.^[6]^ These drugs offer benefits in terms of alleviating symptoms associated with overall changes in cognition, function, behavior, and clinical outcomes.^[7–10]^ There may also be increased risk of adverse events (AE) associated with the use of these medications. For instance, acetylcholinesterase inhibitors like Donepezil, when administered higher doses of for AD, may elevate the risk of bradycardia.^[11]^ Additionally, the concurrent use of other medications may further heighten the risk of AE.^[12]^

However, assessments of the efficacy in AD usually often prioritize short-term cognitive improvement rather than on long-term outcomes.^[13,14]^ To ascertain the long-term efficacy of drugs, a systematic review and analysis of pairwise and network meta-analyses are conducted.

The network meta-analysis (NMA) synthesizes both direct and indirect evidence from network trials comparing multiple interventions.^[15]^ This method enables the comparison of all available AD drugs with placebo and facilitates comparisons between different drug pharmacologies, even in absence of direct a comparison in randomized controlled trials (RCTs). In summary, our aim is to conduct a systematic review and network meta-analysis to compare the efficacy and safety of various drugs, thereby offering improved treatment options for patients with AD over the long-term (>1 year). More importantly, this study aims to provide clinicians with an initial reference for selecting drugs in the long-term treatment of AD.

## 2. Methods

This study constitutes a systematic review and network meta-analysis of RCTs focusing on pharmacological intervention for AD. We prospectively registered the network meta-analysis in PROSPERO with the registration number CRD42021270901. We adhered the Preferred Reporting Items for Systematic Reviews and Meta-Analyses extension statement for network meta-analyses^[16]^ throughout our study.

### 2.1. Search strategy

The following electronic databases were searched from inception to July 30, 2023: PubMed, EMBASE, Web of Science, the Cochrane Central Register of Controlled Trials, Scopus and CNKI. The search terms included medical subject headings terms as well as Title/Abstract terms. For instance, terms such as “Alzheimer’s disease” and specific drug names like “Donepezil,” “Rivastigmine,”and “Galantamine” were used (Table S1, Supplemental Digital Content, http://links.lww.com/MD/N619 which illustrates the search strategy). Additionally, the reference lists of searched reviews and trials were also screened to identify potentially related studies.

### 2.2. Inclusion and exclusion criteria

#### 2.2.1. Types of studies

Eligible RCTs encompassed placebo controls and any active pharmacological intervention, either alone or in combination. Studies lacking full text or presented as case reports were excluded from consideration. The language of the RCTs was limited to English and Chinese.

#### 2.2.2. Types of participants

Participants aged over 18 were diagnosed with AD according to the criteria outlined in the Diagnostic and Statistical Manual of Mental Disorders or the National Institute of Neurological and Communicative Disorders and Stroke and the Alzheimer’s Disease and Related Disorders Association.^[17]^ Participants were excluded if they had been diagnosed with other types of dementia, such as vascular dementia.

#### 2.2.3. Types of interventions

The intervention comprised any drug approved for the treatment of AD. The comparator consisted of either a placebo or any drug used to treat AD, either alone or in combination. There were no restrictions placed on the form or dose of administration.

#### 2.2.4. Types of outcome measures

Outcome measures included 1 or more of the following: function, cognitive, behavior, and AE. The follow-up period for included was a minimum of 1 year. The primary outcome was cognition, assessed using the Alzheimer’s Disease Assessment Scale-cognition subscale.^[18]^ The second outcomes including the flowing: function was measured by Alzheimer’s Disease Cooperative Study activities of daily living scale^[19]^ or the Disability Assessment in Dementia^[20]^; behavior was measured by the neuropsychiatric inventory^[21]^; AE included nausea and/or vomiting, diarrhea, dizziness, loss of appetite and/or anorexia, headache and hypertension. If the number of AE was not reported, the number of participants who experienced any AE (one or more) was used in a study.

### 2.3. Study selection

This review strictly adhered to a three-stage screening method to select relevant RCTS. Firstly, all irrelevant topics and papers screened and excluded by a reviewer (XYD). Secondly, 2 reviewers (XYD and DSL) independently reviewed the title and abstract of each study. Thirdly, 2 reviewers (XYD and DSL) evaluated the full text of each potentially eligible study against the eligibility criteria independently. If disagreements arise, they will be resolved through discussion with consultation between the reviewers.

### 2.4. Data collection

Two authors (XYD and DSL) independently screened the trials to be included in the network meta-analysis and extracted data from studies using a standardized format. The extracted information including the author, year of publication, country, population characters (sample size, baseline mean age, male-to-female ratio, diagnostic criteria, interventions, follow-up period, and outcomes). The first author proofread for accuracy. To obtain missing data, we emailed the author or estimated it using available such as means, standard deviations, standard errors, confidence interval (CI) or *P* values.^[22]^ All discrepancies were resolved through discussions with the authors.

### 2.5. Quality assessment

One author (XYD) assessed the risk of bias, a second author (DSL) checked the assessment, and another author reviewed (DSL) the final results and resolved any differences. We used the Cochrane Collaboration’s tool^[23]^ to evaluate the risk of bias in eligible studies base on 7 domains (random sequence generation, allocation concealment, blinding of participants, blinding of outcome assessment, incomplete outcome data, and selective outcome reporting). We provide comprehensive descriptions of all judgements and presented our conclusions in the “Risk of bias” figures. The risk of bias in each domain can be judged to be high, unclear, or low.

### 2.6. Synthesis

The pooled effect sizes were represented using standardized mean different or mean different (MD) for continuous outcomes, and odds ratios (ORs) for dichotomous outcomes, accompanied by their corresponding 95% CI. Firstly, we conducted pair-wise meta-analyses by combining included studies that compared the same interventions. We utilized the *I*^2^ to evaluate the heterogeneity of direct comparison in the analysis. *I*^2^ > 50%, random-effects model was employed. *I*^2^ < 50%, fixed-effects model was used. Begg and Egger regression test were employed to examine publication bias in the direct comparison. Stata software (version 15.0) was used to analysis this direct meta-analysis. Sensitivity analysis was performed to verify the stability of the results.

Secondly, Bayesian network meta-analysis combining both direct and indirect comparisons based on a random-effect model, was conducted. Network meta-analysis (NMA) is a method synthesize information from a network of trials that address the same question but involve different interventions. NMA combines direct and indirect evidence across a network of randomized trials into a single effect size and, under certain assumptions, can enhance the precision of the estimates while adhering to randomization principles. The model allowed us to estimate the probability that each intervention is the best for each outcome, given the relative effect sizes as estimated in NMA. GeMTC (version 14.3) and Stata (version16.0) were utilized to perform the analysis. In the case of three Markov chains running simultaneously, different initial values are randomly selected. The total number of iterations is 30,000. The CI included 0 or 1 was used to assess statistical significance. The intervention hierarchy was estimated using the surface under the cumulative ranking curve (SUCRA). The SUCRA metric was employed to rank the effectiveness of each treatment and identify the best treatment. An advantage of NMA is its capability to offer a coherent ranking of treatments, with the SUCRA. SUCRA values range between 0% and 100%, with higher values indicating a greater likelihood that the treatment is ranked at the top.^[24]^ In addition, statistical inconsistency was evaluated using the node-splitting method. The significance of loop consistency relies on the CI value of the inconsistency factor (IF) value being 0.

## 3. Results

**Figure 1.**
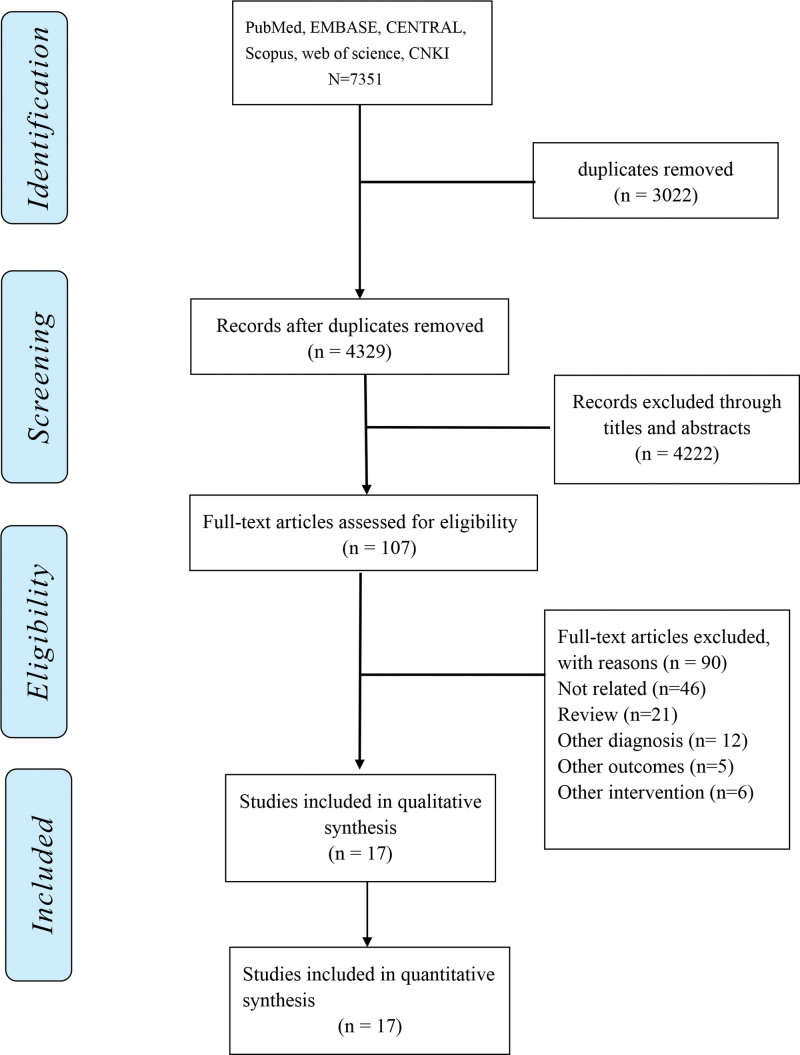
PRISMA flowchart. PRISMA = Preferred Reporting Items for Systematic Reviews and Meta-Analyses.

The search identified a total of 7351 records. After excluding 3022 duplicates, 107 records remained following the review of the abstracts or titles. After reading full text, 17 records met the inclusion criteria (Fig. 1). There were 7 pharmacological interventions in these RCTs, including Donepezil, Rivastigmine, Galantamine, Memantine, Ginkgo biloba extract, Atorvastatin-calcium, and Vitamin B. Ten of the included were placebo-controlled trials. The characteristics of the studies were presented in Table 1. The included studies comprised 7214 patients, with a mean age ranging from 64 to 80 years.

**Table 1:** Characteristics of studies.

Study	No of patients	Gender (% female)	Age mean (SD), year	Treatment and dose	Diagnostic criteria	Follow-up	Outcomes/efficacy measures
Le Bars PL^[25]^ 1997 USA	120	65/54	68 (10)	EGb 120 mg	DSMIII-R ICD-10	52 weeks	ADAS-cog GERRI CGIC
	116	72/62	68 (11)	Placebo			
Farlow M^[26]^ 2000 USA	125	NA	74.3	Rivastigmine 12 mg	NA	52 weeks	ADAS-cog
	151	NA	74.3	Rivastigmine 4 mg			
	144	NA	74.3	Placebo			
Winblad B^[27]^ 2001 Sweden	142	99/69.7	72.1 (8.6)	Donepezil 10 mg	NINCDS-ADRDA	52 weeks	MMSE GDS
	144	85/59.0	72.9 6 (8.0)	placebo			
Mohs RC^[28]^ 2001 USA	214	131/61.2	75.4 (0.6)	Donepezil 10 mg	DSM-IV NINCDS	12 months	ADFACS MMSE CDR
	217	140/64.5	75.3 (0.6)	Placebo			
Le Bars PL^[29]^ 2002 USA	MMSE > 23						
	61	34/56	64 (9)	Ginkgo biloba extract 120 mg	ICD-10 DSM-III-R	52 weeks	ADAS-cog GERRI CGIC
	61	38/62	64 (11)	Placebo			
	MMSE < 24						
	59	31/53	73 (9)	Ginkgo biloba extract 120 mg			
	55	34/62	72 (9)	Placebo			
Bullock R^[30]^ 2005 USA	495	341/68.9	75.9 (6.6)	Rivastigmine 12 mg	DSM-IV NINCDS-ADRDA	104 weeks	SIB GDS ADCS–ADL MMSE NPI
	499	342/68.5	75.8 (6.8)	Donepezil 10 mg			
Karaman Y^[31]^ 2005 Turkey	24	13/54.1	74.11 (0.87)	Rivastigmine 12 mg	NINCDS-ADRDA DSM-IV	1 year	ADAS-cog CIBIC-plus MMSE PDS ADCD/ADL DAD GDS
	20	11/55	73.4 (0.9)	Placebo			
Sparks DL^[32]^ 2005 USA	32	12/37.5	78.15 (1.3)	Atorvastatin calcium 80 mg	NINCDS-ADRDA	12 months	ADAS-cog CGIC NPI MMSE GPS ADCS-ADL
	31	11/35.5	78.9 (1.2)	Placebo			
Bullock R^[33]^ 2006 USA	Age < 75 years				NINCDS-ADRDA DSM-IV	24 months	SIB NPI-10 NPI-D GDS MMSE ADCS-ADL
	177	114/64.4	68.8 (5.2)	Rivastigmine 12 mg			
	185	121/65.4	68.8 (5.4)	Donepezil 10 mg			
	Age > 75 years						
	318	227/71.4	79.8 (3.1)	Rivastigmine 12 mg			
	314	221/70.4	80 (3.2)	Donepezil 10 mg			
Aisen PS^[6]^ 2008 USA	240	138/57.5	75.7 (8.0)	Vitamin B	NINCDS-ADRDA	18 months	ADAS-cog CDR NPI ADCS-ADL
	169	91/53.9	77.3 (7.9)	Placebo			
Haihua H 2011, China	32	18/56	72.44 (4.67)	Memantine 20 mg	DSM-IV NINCDS-ADRDA	12 months	MMSE ADAS-cog ADL GDS
	32	16/50	71.78 (3.35)	Rivastigmine 9 mg			
Haihua H 2011, China	28	12/42	72.18 (4.35)	Rivastigmine 9 mg + EGb120 mg	DSM-IV NINCDS-ADRDA	12 months	MMSE ADAS-cog ADL GDS
	28	14/50	71.29 (3.36)	EGb 120 mg			
Baoli Z^[34]^ 2013 China	52	24/46	69. 7 (5.4)	Memantine 20 mg + EGb 120 mg	DSM-IV NINCDS-ADRDA	12 months	MMSE ADL
	52	25/48	69. 9 (5.5)	EGb 120 mg			
Wilkinson D^[35]^ 2012 UK	133	83/62	74 (9)	Memantine 20 mg	NINCDS-ADRDA	52 weeks	COWAT, CFT ADAS-cog Stroop C StroopI MMSE, NPI
	144	75/52	74 (8)	Placebo			
Hager K^[36]^ 2014, USA	1024	671/65.5	73 (8.9)	Galantamine 18 mg	NINCDS-ADRDA	24 months	MMSE, DAD
	1021	654/64.1	73 (8.7)	Placebo			
Peters O^[37]^ 2015 Germany	112	76/68.4	72.1 (8.5)	20 mg memantine + 24 mg galantamine	NINCDS-ADRDA The German Dementia Competence Network	52 weeks	ADAS-cog ADCS-ADL CDR
	114	68/58.9	72.6 (7.8)	24 mg galantamine			
Yokoyama S^[38]^ 2019 Japan	37	12/25	77.0 (6.8)	Donepezil 5 mg + placebo	DSM-IV	12 months	ADAS-J cog MMSE NPI GDS
	42	15/27	76.1 (7.6)	Donepezil 5 mg + teprenone 300 mg			

ADAS-cog12 = Alzheimer’s Disease Assessment Scale-Cognitive Subscale, ADAS-J cog = the Japanese version of the AD Assessment Scale-cognitive subscale, ADCS-ADL = the Alzheimer’s Disease Cooperative Study-Activities of Daily Living score, ADCS-IADL = Alzheimer’s Disease Cooperative Study Activities of Daily Living inventory, instrumental items; TMT-A and TMT-B the Trail Making Test parts A and B, CDR-SB = Clinical Dementia Rating-Sum of Boxes scores, CGIC = Clinical Global Impression of Change Scale, CIBIC-Plus = the Clinicians Interview Based Impression of Change Plus, DAD = the Disability Assessment in Dementia, DSM-IV = Diagnostic and Statistical Manual of Mental Disorders, Fourth Edition, EGb = Ginkgo biloba extract, GDS = Global Deterioration Scale, GDS = the Progressive Deterioration Scale, GERRI = the Geriatric Evaluation by Relative’s Rating Instrument, GPS = Geriatric Depression Scale, ICD-10 = International Statistical Classification of Diseases, 10th Revision criteria, MMSE = Mini-Mental State Examination, NINCDS-ADRDA = the National Institute of Neurological and Communicative Disorders and Stroke and the Alzheimer’s Disease and Related Disorders Association, NPI-10 = the 10-item Neuropsychiatric Inventory, NPI-D = the NPI-caregiver distress scale, SADAS-cog = Standardized Alzheimer’s Disease Assessment Scale cognitive subscale, SIB = Severe impairment Battery.

### 3.1. Risk of bias

In most trials random sequence generation was employed. In 9 trials, adequate allocation concealment was considered as low risk of bias. In 7 trials, outcome assessors were blinded. In 14 trials, incomplete outcome data were considered as low risk of bias. In 15 trials, selective reporting was considered as low risk of bias (Fig. S1, Supplemental Digital Content, http://links.lww.com/MD/N620 and Fig. S2, Supplemental Digital Content, http://links.lww.com/MD/N620, which illustrates the risk of bias).

### 3.2. Pairwise meta-analysis

#### 3.2.1. Cognition

For cognition, changes longer than 1 year were assessed using ADAS-cog. Ten trials^[6,25,26,29,31,32,34,37–40]^ were included in this analysis. There was no statistically significant difference between the drugs, except for the finding that rivastigmine 12 mg + Ginkgo biloba extract (EGb) 120 mg improved cognition more effectively than EGb 120 mg alone (MD = 9.27, CI 2.76, 15.78). Atorvastatin calcium 80 mg was found improve cognition more effectively than placebo (MD = 6.93, CI 6.07, 7.79) (Table 2).

**Table 2:** Results of pairwise meta-analysis.

Compare	Outcomes	Effect	95% CI	*P*
	Cognition-ADAS-cog			
Rivastigmine 12 mg vs placebo		3.18	[‐0.91, 7.27]	.13
EGb 120 mg vs placebo		‐0.83	[‐3.78, 2.13]	.58
Donepezil 5 mg + teprenone 300 mg vs donepezil 5 mg		‐0.20	[‐2.41, 2.01]	.86
Rivastigmine 12mg + EGb120 mg vs EGb120mg		9.27	[2.76, 15.78]	.005*
Rivastigmine 13.3 mg vs rivastigmine 9.5 mg		2.30	[0.95, 3.65]	.0008*
Rivastigmine 12 mg vs memantine 20 mg		1.67	[‐3.58, 6.92]	.53
Memantine 20 mg + galantamine 24 mg vs galantamine 24 mg		0.47	[‐0.00, 0.94]	.05
Vitamin b vs placebo		‐0.04	[‐1.31, 1.23]	.95
Atorvastatin calcium 80 mg vs placebo		6.93	[6.07, 7.79]	<.00001*
	Function-ADCS-ADL			
Rivastigmine 12 mg vs placebo		‐0.18	[‐0.27, −0.09]	.0001*
Rivastigmine 12 mg + EGb 120 mg vs EGb 120 mg		9.11	[2.48, 15.74]	.007*
Memantine 20 mg vs rivastigmine 9 mg		2.03	[‐2.87, 6.93]	.42
Rivastigmine 13.3 mg vs rivastigmine 9.5mg		2.30	[0.95, 3.65]	.0008*
Memantine 20 mg + galantamine 24 mg vs galantamine 24 mg		‐2.64	[‐3.39, −1.89]	<.00001*
Memantine 20 mg + EGb 120 mg vs EGb 120mg		‐13.20	[‐16.58, −9.82]	<.0001*
Rivastigmine 12 mg vs Donepezil 10 mg		0.82	[‐0.39, 2.03]	.19
Vitamin B vs placebo		0.44	[‐1.53, 2.41]	.66
	Behavior-NPI			
Donepezil 5 mg combined teprenone 300 mg vs donepezil 5 mg		‐1.20	[‐3.21, 0.81]	.24
Rivastigmine 12 mg vs donepezil 10 mg		‐0.57	[‐5.05, 3.91]	.80
Rivastigmine 13.3 mg vs rivastigmine 9.5 mg.		0.50	[‐1.58, 2.58]	.64
Vitamin B vs placebo		‐0.16	[‐2.10, 1.78]	.87
Atorvastatin calcium 80 mg vs placebo		‐4.30	[‐6.01, −2.60]	<.0001*
	Safety			
Donepezil 10 mg vs placebo		1.08	[0.96, 1.22]	.22
EGb 120 mg vs placebo		1.11	[1.02, 1.21]	.01*
Rivastigmine 12 mg vs placebo		1.20	[0.64, 2.25]	.57
Donepezil 5 mg combined teprenone 300 mg vs donepezil 5 mg		0.66	[0.25, 1.73]	.40
Memantine 20 mg combined galantamine 24 mg vs galantamine 24 mg		1.06	[0.63, 1.80]	.82
Rivastigmine 12 mg and donepezil 10 mg		1.26	[1.20, 1.34]	<.0001*
Galantamine 24 mg vs placebo		1.11	[1.02, 1.21]	.01*
Rivastigmine 12 mg combined EGb 120 mg vs EGb120 mg		19.00	[1.16, 310.37]	.04*
Memantine 20 mg vs rivastigmine 9 mg		0.58	[0.36, 1.76]	.77
Rivastigmine 13.3 mg vs rivastigmine 9.5 mg		1.02	[0.93, 1.13]	.50

ADAS-cog = Alzheimer’s Disease Assessment Scale-cognition subscale, ADCS-ADL = Alzheimer’s Disease Cooperative Study activities of daily living scale, EGb = Ginkgo biloba extract, NPI = neuropsychiatric inventory.

* The comparison was statistically significant.

#### 3.2.2. Function

For function, changes longer than 1 year were assessed using Alzheimer’s Disease Cooperative Study activities of daily living. Eight trials^[6,30,31,33,34,37,39,40]^ were included to analysis. There was no statistically significant difference between the drugs except for the following findings: Rivastigmine 12 mg was better than placebo (MD = ‐0.18, CI −0.27, −0.09); Memantine 20 mg + Galantamine 24 mg was better than Galantamine 24 mg (MD = ‐2.64, CI −3.39, −1.89); Memantine 20 mg + EGb 120 mg were better than EGb 120 mg (MD = ‐13.20, CI −3.39, −1.89); Rivastigmine 12 mg + EGb 120 mg were better than EGb 120 mg (MD = 9.11,CI 2.48, 15.74) (Table 2).

#### 3.2.3. Behavior

For behavior, changes longer than 1 year were assessed using neuropsychiatric inventory. Five trials^[6,30,32,33,38]^ were included to analysis. There was no statistically significant difference between the drugs, except for Atorvastatin calcium 80 mg was better than placebo (MD = ‐4.30, CI ‐6.01, ‐2.60) (Table 2).

#### 3.2.4. Safety

For safety, 13 trials^[26–28,30,31,33,35–40]^ were included in the analysis. At this follow-up period, statistically significant differences were observed between the following comparisons: EGb 120 mg and placebo, in favor of placebo (OR = 1.11, CI 1.02, 1.21); Rivastigmine 12 mg and Donepezil 10 mg, in favor of Donepezil 10 mg (OR = 1.26, CI 1.20, 1.34); Rivastigmine 12 mg + EGb 120 mg and EGb120 mg, in favor of EGb 120 mg (OR = 19, CI 1.16, 310.37); Galantamine 24 mg and placebo, in favor of placebo (OR = 1.11,CI 1.02, 1.21) (Table 2).

### 3.3. Network meta-analysis

#### 3.3.1. Cognition

**Figure 2.**
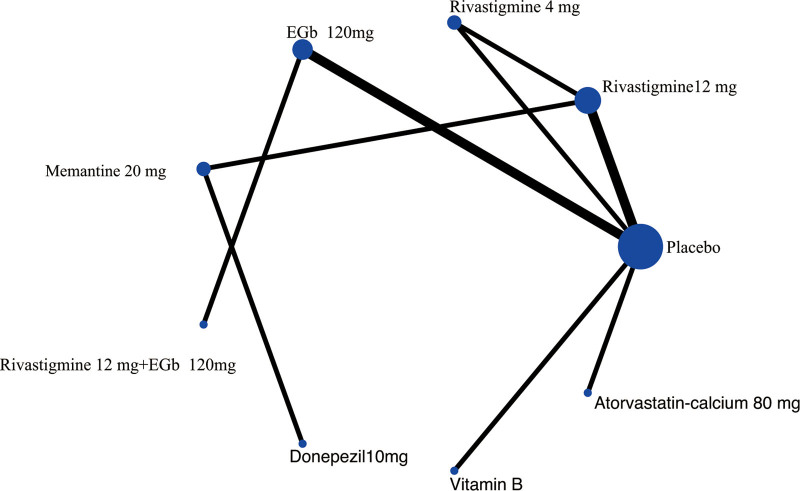
The network plot for cognition based on ADAS-cog. ADAS-cog = the Alzheimer’s Disease Assessment Scale-cognition subscale.

**Figure 3.**
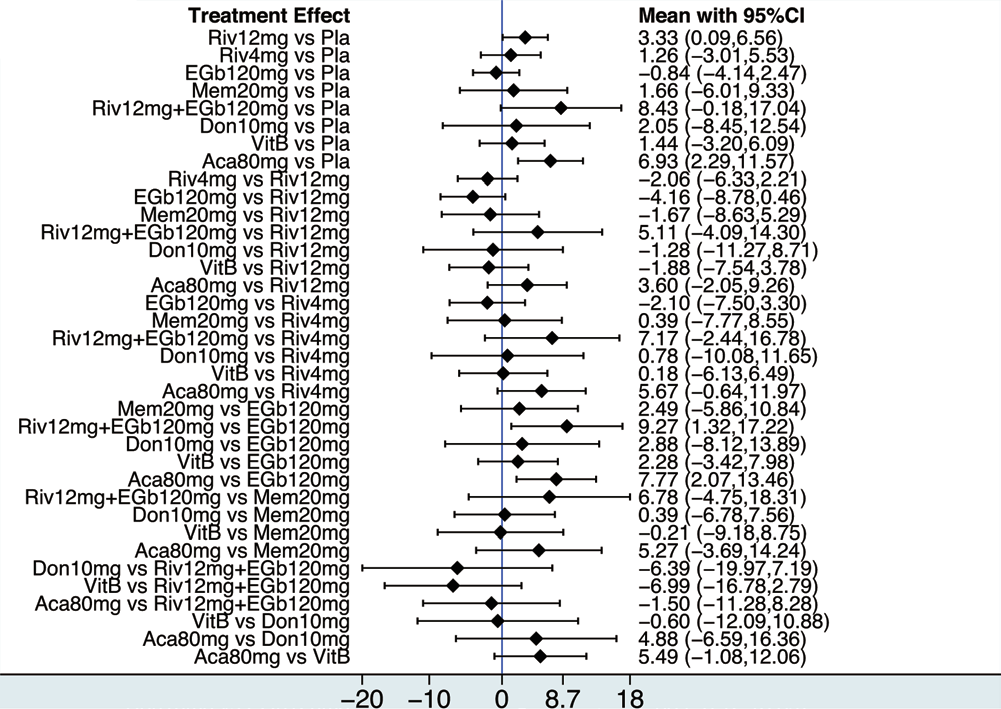
The forest plot for cognition based on ADAS-cog. ADAS-cog = the Alzheimer’s Disease Assessment Scale-cognition subscale.

**Figure 4.**
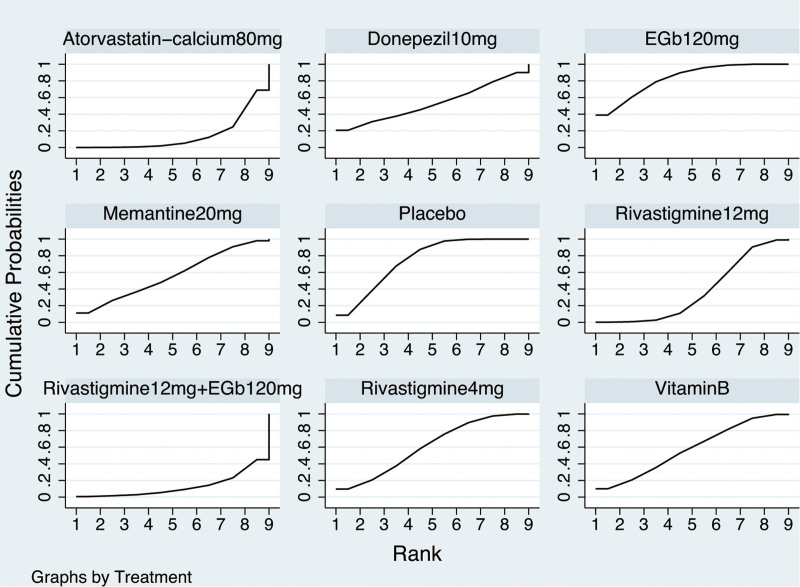
Ranking for cognition based on ADAS-cog. ADAS-cog = the Alzheimer’s Disease Assessment Scale-cognition subscale.

The NMA on the ADAS-cog comprised 9 RCTs involving 1468 participants. The network diagram was presented in Figure 2. Six interventions were directly compared with the placebo. There was 1 closed loop, indicating both direct and indirect comparisons enhancing the reliability of the conclusions, across all comparisons. Based on the results of the IFs and 95% CIs, the evidence was consistent (Fig. S3, Supplemental Digital Content, http://links.lww.com/MD/N620 which illustrates the consistent). The results indicated that Atorvastatin-calcium 80 mg was superior to placebo (MD = ‐6.93, CI −11.57, −2.29), and Rivastigmine 12 mg was superior to placebo (MD = ‐3.33, CI −6.56, −0.09). EGb120 mg was found to be superior to Atorvastatin-calcium 80 mg (MD = 7.77, CI 2.07, 13.46) and Rivastigmine 12 mg + EGb120 mg (MD = 9.27, CI 1.32, 17.22) (Table 3). The forest plot was present at Figure 3. Base on the SUCRA, the most efficient intervention was ranked as EGb 120 mg (83%) (Fig. 4). The funnel plot was seen in Figure S4, Supplemental Digital Content, http://links.lww.com/MD/N620 which illustrates the funnel plot.

**Table 3:** Results of network meta-analysis on cognition.

Placebo								
**‐6.93 (‐11.57, ‐2.29**)	AC 80 mg							
‐1.44 (‐6.09, 3.20)	5.49 (‐1.08, 12.06)	Vitamin B						
‐2.05 (‐12.54, 8.45)	4.88 (‐6.59, 16.36)	‐0.60 (‐12.09, 10.88)	Donepezil 10 mg					
‐8.43 (‐17.04, 0.18)	‐1.50 (‐11.28, 8.28)	‐6.99 (‐16.78, 2.79)	‐6.39 (‐19.97, 7.19)	R 12 mg + EGb 120mg				
‐1.66 (‐9.33, 6.01)	5.27 (‐3.69, 14.24)	‐0.21 (‐9.18, 8.75)	0.39 (‐6.78, 7.56)	6.78 (‐4.75, 18.31)	Memantine 20 mg	–		
0.84 (‐2.47, 4.14)	**7.77 (2.07, 13.46**)	2.28 (‐3.42, 7.98)	2.88 (‐8.12, 13.89)	**9.27 (1.32, 17.22**)	2.49 (‐5.86, 10.84)	EGb 120 mg		
‐1.26 (‐5.53, 3.01)	5.67 (‐0.64, 11.97)	0.18 (‐6.13, 6.49)	0.78 (‐10.08, 11.65)	7.17 (‐2.44, 16.78)	0.39 (‐7.77, 8.55)	2.49 (‐5.86, 10.84)	Rivastigmine 4 mg	
**‐6.93 (‐11.57, ‐2.29**)	3.60 (‐2.05, 9.26)	‐1.88 (‐7.54, 3.78)	‐1.28 (‐11.27, 8.71)	5.11 (‐4.09, 14.30)	‐1.67 (‐8.63, 5.29)	0.39 (‐7.77, 8.55)	‐2.06 (‐6.33, 2.21)	Rivastigmine 12 mg

Bold values mean MDs are statistically significant.

AC = Atorvastatin-calcium; R + EGb = Rivastigmine12 mg + Ginkgo biloba extract 120 mg.

#### 3.3.2. Safety

**Figure 5.**
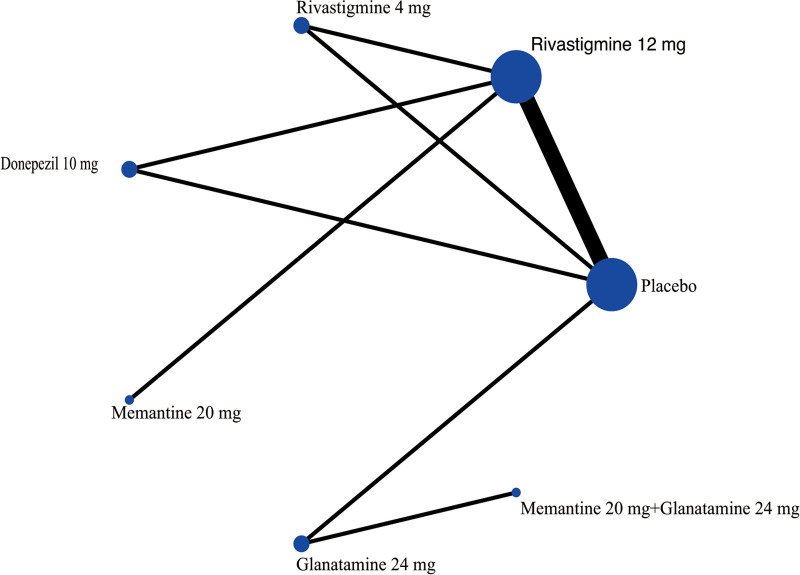
The network plot for safety based on adverse events.

**Figure 6.**
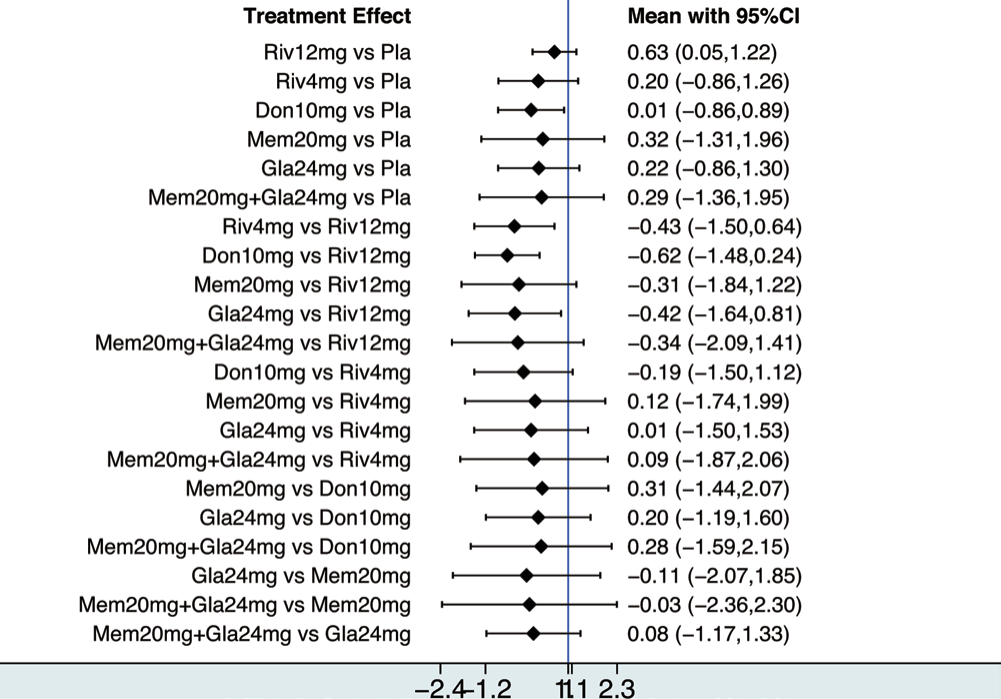
The forest plot for safety based on adverse events.

**Figure 7.**
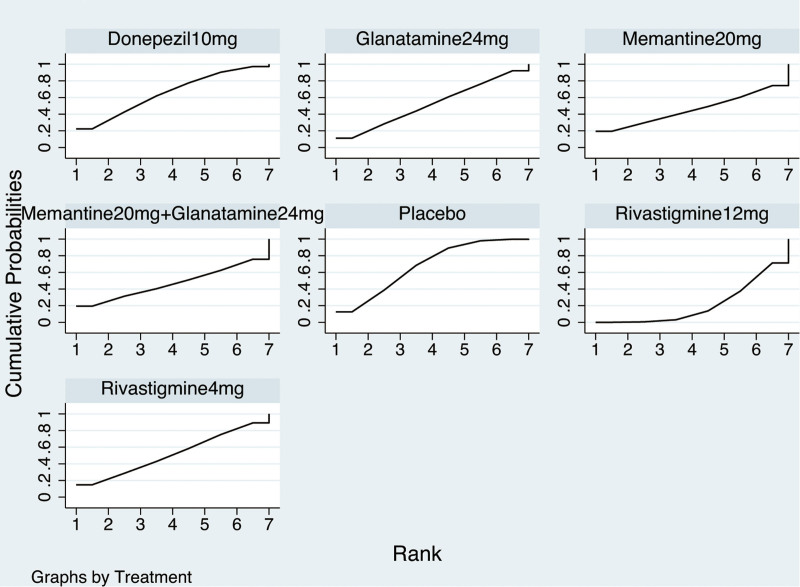
Ranking for cognition based on adverse events.

The NMA of AE included 9 RCTs with 5185 participants. The network diagram was presented in Figure 5. Six interventions were directly compared with the placebo. There were 2 closed loops existing across all comparisons. Based on the results of the factor (IFs) and 95% CIs, the evidence was consistent (Fig. S5, Supplemental Digital Content, http://links.lww.com/MD/N620 which illustrates the consistency). There was no significantly difference between interventions (Table 4). The forest plot was present at Figure 6. Based on SUCRA, placebo (67.8%) was most likely to be ranked first (Fig. 7). The funnel plot was seen in Figure S6, Supplemental Digital Content, http://links.lww.com/MD/N620 which illustrates the funnel plot.

**Table 4:** Results of network meta-analysis on adverse events.

Placebo						
‐0.29 (‐1.95, 1.36)	Memantine 20 mg + Galantamine 24 mg					
‐0.22 (‐1.30, 0.86)	0.08 (‐1.17, 1.33)	Galantamine 24 mg				
‐0.32 (‐1.96, 1.31)	‐0.03 (‐2.36, 2.30)	‐0.11 (‐2.07, 1.85)	Memantine 20 mg			
‐0.01 (‐0.89, 0.86)	0.28 (‐1.59, 2.15)	0.20 (‐1.19, 1.60)	0.31 (‐1.44, 2.07)	Donepezil 10 mg		
‐0.20 (‐1.26, 0.86)	0.09 (‐1.87, 2.06)	0.01 (‐1.50, 1.53)	0.12 (‐1.74, 1.99)	‐0.19 (‐1.50, 1.12)	Rivastigmine 4 mg	
‐0.63 (‐1.26, 0.05)	‐0.34 (‐2.09, 1.41)	‐0.42 (‐1.64, 0.81)	‐0.31 (‐1.84, 1.22)	0.62 (‐1.48, 0.24)	‐0.43 (‐1.50, 0.64)	Rivastigmine 12 mg

## 4. Discussion

In the systematic review and network meta-analysis with long term (>1 year) RCTs included, 7214 participants were included. In pairwise meta-analysis, Rivastigmine 12 mg + EGb 120 mg and Atorvastatin calcium 80 mg showed benefits for cognition. Rivastigmine 12 mg, Memantine 20 mg + Galantamine 24 mg, Memantine 20 mg + EGb 120 mg, Rivastigmine 12mg + EGb 120 mg, and Rivastigmine 12 mg + EGb 120 mg were beneficial for function. Atorvastatin calcium 80 mg was beneficial for behavior. The placebo, Donepezil 10 mg, and EGb 120 mg were beneficial for safety. In network meta-analysis, placebo and EGb 120 mg were beneficial for cognition, with placebo being the least harm over the long-term (>1 year). Some published systematic reviews have investigated the effects of drugs on AD. Tricco, AC et al^[41]^ demonstrated that Donepezil + Memantine is the most-effective therapy for AD, followed by Donepezil and Galantamine. The results were inconsistent with ours. Here are some possible reasons for the inconsistency in the results. Firstly, they initially included RCTs, non-RCTs, and cohort studies. However, we included only RCTs. Secondly, they did not prioritize flow-ups, whereas we focused on flow-ups longer than 12 months. Zhang, T et al^[8]^ suggested that galantamine 32 mg, galantamine 24 mg, Donepezil 5 mg, Donepezil 10 mg, and Memantine 20 mg lead to better cognitive improvement than other medications in patients with mild to moderate AD. Veroniki, A. A. et al^[42]^ identified Donepezil + Memantine, Donepezil alone, and transdermal Rivastigmine as the most effective treatments for improving MMSE scores. Oral Rivastigmine and Donepezil had the least favorable safety profiles. For moderate-to-severe impairment, Donepezil, Memantine and their combination improved MMSE scores, when adjusted for comorbidities, only oral Rivastigmine was effective. For mild-to-moderate impairments, Donepezil and transdermal Rivastigmine improved MMSE scores. The longest follow-up period in their study was 24 weeks. These are inconsistent with our findings. The primary difference likely stems from variations in follow-up duration and the predominance of participants with mild-moderate AD in most studies. However, our study did not differentiate based on stage of AD. Lyu, D. et al^[43]^ designed systematic review and network meta-analysis protocols focusing on the cognitive benefits of various drug categories, such as anti-beta-amyloid, anti-Tau, metabolism, symptomatic, etc. These protocols have the potential to uncover key factors in treating AD and preserving cognitive function. Through Bayesian network meta-analysis, they aim to rank the effects of different hypotheses on enhancing cognitive function and treating AD. Their findings are expected to contribute to drug development and inform clinical practice. However, our study did not classify the drugs for AD, but simply studied the effects of each drug on AD. EGb761 240 mg appears to be optimal in terms of acceptability and safety. The results are also inconsistent with ours. They collected data from baseline to final point, which included various timeframes, but we included flow-up periods longer than 12 months. The study^[44]^ by Sun, Yuan et al showed insufficient evidence to recommend Atorvastatin calcium for mild to moderate AD. There was no benefit observed for outcome measures of general function, cognitive function, or mental/behavioral abnormalities. This findings contradicts, which suggests that Atorvastatin calcium may have some effect on dementia. Excess cholesterol in the brain promotes amyloid production, while Atorvastatin calcium may lower cholesterol levels. At the same time, Atorvastatin calcium also exhibit anti-inflammatory effects. But our study includes only 1 trial, so the efficacy needs to be confirmed by larger, higher-quality randomized controlled trials, especially for moderate-to-severe AD.

Based on the evidence, we suggest that EGb 120 mg and placebo may be considered as the treatment for AD for long-term cognition improvement, with placebo showing the least harm to patients with AD. Current studies have found that the total flavonoids in Ginkgo biloba leaves have antioxidant,^[45]^ anticancer^[46]^ and antibacterial^[47]^effects. Ginkgo biloba is a natural human thrombin inhibitor that can prevent and treat thrombosis and cardiovascular disease.^[48]^ Ginkgolide has the function of regulating neurotransmitter and neuromodulator.^[49]^ In addition, Ginkgolide B, as the most effective platelet-activating factor receptor antagonist, exhibits antithrombotic and anti-inflammatory activities.^[50]^ EGb partially improves memory and cognitive impairment caused by AD by modulating synaptic plasticity. After 1 month of intraperitoneal injection of ginkgo biloba extract in transgenic mice with 5 familial AD, the hippocampus dentate gyrus showed an increase in the number of new neurons, and the spatial and nonspatial memory of the model mice were improved.^[51]^ AD is a neurodegenerative disease characterized by the formation of senile plaques, composed of neuronal tangles formed from aggregated beta-amyloid protein fibers and hyperphosphorylated Tau protein (p-Tau).^[52]^ Studies have shown that autophagy activation of Tau transgenic mice through oral administration of EGb facilitates the removal of p-Tau protein and ameliorates synaptic damage caused by Tau protein.^[53]^ Increasing Protein Phosphatase 2A activity in AD brain can mitigate the hyperphosphorylation of Tau and amyloid precursor protein, consequently decreasing the formation of amyloid plaques lake and p-Tau.^[54]^ Ginkgo biloba leaves primarily contain 2 active components: flavonoid glycosides and terpene lactones (including ginkgolides such as ginkgolides A, B, etc). Our study demonstrated that the placebo had the least harm effects on AD. This could be attributes various safety and tolerance issues, such as infection, asymptomatic lacunar infarction, alcoholism, and knee osteoarthritis, among others. The extended duration of study may have a role in the placebo’s comparative safety compared to the drug. Further studies will be necessary to substantiate, and we will continue to update our findings with new literature to obtain more scientifically robust results.

To mitigate bias risks, future studies should adopt stringent methodology and consider follow-up periods exceeding 12 months. Future studies should incorporate homogeneous populations, including groups with mild and severe AD, characterized by common clinical manifestation, diagnostic criteria, and duration of symptoms. Additionally, there is a need for health economic evaluations to assess the cost-effectiveness of interventions.

### 4.1. Strengths and limitations

We included studies with long-term follow-up (>1 year) and conducted a comprehensively comparison of the effects and safety profiles of various drugs. To our knowledge, our study is the first to conduct a long-term (>1 year) systematic review of treatment for AD and perform network meta-analysis. Our findings regarding the long-term drug treatment of AD may offer valuable insights for clinicians in selecting appropriate medications. We adhered to the assigned protocol to conduct the study, utilizing the latest recommendations to assess the studies ``bias risk’’. But there were some limitations. Firstly, data were pooled from follow-up after 1 year, but some studies included data from periods longer than 1 year. Secondly, some studies did not provide all outcome indicators of interest, resulting in incomplete pooled results. Thirdly, a SUCRA curve was used to estimate the ranking of the probability of comparison between different treatment effectiveness, but it has limitations. Therefore, the results should be carefully explained. Fourthly, most of the studies fails to detail the precise chemical composition of the placebo, a factor that can impact result consistency. Fifthly, the limited availability evidence results in number of studies on Atorvastatin calcium and Vitamin B, leading to inherent limitations. Finally, our study is limited to English and Chinese languages, potentially excluding pertinent literature in other languages, which could result in overlooking crucial studies.

## 5. Conclusion

In summary, our network meta-analysis indicates that EGb 120 mg is benefit for cognition over a period longer than 1 year, while placebo showed the least harm. The estimates of effect size for change in all comparisons were uncertain. Additional RCTs are required to clarify the efficacy of treatment for AD.

## Acknowledgments

We are grateful for the partnership and support from Sichuan Provincial People’s Hospital Library for the development of search strategy.

## Author contributions

**Conceptualization:** Xiaoyan Deng.

**Data curation:** Xiaoyan Deng.

**Funding acquisition:** Daishun Li.

**Investigation:** Xiaoyan Deng, Daishun Li.

**Methodology:** Daishun Li.

**Software:** Xiaoyan Deng.

**Writing – original draft:** Xiaoyan Deng.

**Writing – review & editing:** Daishun Li.

## Supplementary Material


