# Supplementary material for: Effect of long-term pharmacological treatments on Alzheimer disease: A systematic review and network meta-analysis
Source: Medicine (Baltimore). 2024 Sep 20;103(38):e39753. doi: 10.1097/MD.0000000000039753 (PMC11419515; doi:10.1097/MD.0000000000039753)
Supplement: Supplementary file 1 [file medi-103-e39753-s001.doc]

**Additional file 1**

## Search strategies for PubMed

| Step | Topic or intervention | Search algorithm | Items found |
| --- | --- | --- | --- |
| #1 | Disease | 'Alzheimer disease'/exp OR 'Alzheimer disease' OR (Alzheimer AND ('disease'/exp OR disease)) OR 'Alzheimer disease': ti, ab, kw OR 'Alzheimer disease': ti, ab, kw  OR 'Alzheimer dement*':ti, ab, kw OR 'Alzheimers dise*':ti, ab, kw OR 'Alzheimer syndr*':ti,ab,kw | 278496 |
| #2 | Study design | 'randomized controlled trial'/exp OR 'randomized controlled trial':ti,ab,it OR 'randomized':ti,ab,it OR 'randomised':ti,ab,it OR 'randomization':ti,ab,it OR 'randomisation':ti,ab,it OR rct:ti,ab,it | 1192617 |
| #3 | Donepezil | 'donepezil'/exp OR 'donepezil' ab,ti OR 'donepezilium oxalatetrihydrate':ab,ti  OR eranz:ab,ti OR 'donepezil hydrochloride':ab,ti  OR aricept:ab,ti | 14092 |
| #4 | Rivastigmine | 'rivastigmine'/exp OR 'rivastigmine tartrate': ab,ti OR 'rivastigmine hydrogen tartrate':ab,ti  OR 'hydrogen tartrate, rivastigmine' :ab,ti  OR 'tartrate, rivastigmine hydrogen’: ab,ti  OR exelon:ab,ti | 7937 |
| #5 | Galantamine | 'galantamine'/exp OR galantamine:ab,ti OR lycoremine:ab,ti OR galantamin:ab,ti OR nivaline:ab,ti OR nivalin:ab,ti OR razadyne:ab,ti OR reminyl:ab,ti OR 'galanthamine hydrobromide':ab,ti | 8169 |
| #6 | huperzine | Huperzine /exp OR huperzine:ab,ti OR 'huperzine a':ab,ti | 1581 |
| #7 | Memantine | 'memantine'/exp OR memantine:ab,ti OR namenda:ab,ti OR ebixa:ab,ti OR 'memantine hydrochloride':ab,ti OR axura:ab,ti | 11659 |
| #8 | nimodipine | 'nimodipine'/exp OR nimodipine:ab,ti  OR brainal:ab,ti OR calnit:ab,ti OR kenesil:ab,ti OR nymalize:ab,ti OR 'nimodipin hexal':ab,ti OR 'nimodipin isis':ab,ti OR 'nimodipino bayvit':ab,ti OR nimotop:ab,ti OR remontal:ab,ti  OR 'admon modus':ab,ti | 11083 |
| #9 | aspirin | ‘aspirin’/exp OR aspirin:ab,ti OR ‘acetylsalicylic Acid’ :ab,ti OR ‘acid, acetylsalicylic’ :ab,ti OR ‘2-(acetyloxy)benzoic acid’ :ab,ti OR acylpyrin :ab,ti OR aloxiprimum :ab,ti OR acetysal :ab,ti OR solupsan:ab,ti OR polopiryna:ab,ti | 23544 |
| #10 | citicoline | 'citicoline'/exp OR citicoline:ab,ti OR 'choline, cytidine diphosphate':ab,ti  OR 'diphosphate choline, cytidine':ab,  OR cidifos:ab,ti OR citicoline:ab,ti  OR 'choline, cdp':ab,ti OR 'cytidine 5-diphosphocholine':ab,ti | 2367 |
| #11 | **vinpocetine** | 'vinpocetine'/exp OR vinpocetine:ab,ti  OR vinpocetine:ab,ti OR kavinton:ab,ti OR cavinton:ab,ti | 1634 |
| #12 | sertraline | sertraline /exp OR sertraline:ab,ti OR ertraline:ab,ti OR zoloft:ab,ti  OR altruline:ab,ti OR 'apo sertraline':ab,ti OR besitran:ab,ti | 346 |
| #13 | **Nafronyl** | 'nafronyl'/exp OR nafronyl:ab,ti OR naftidrofuryl:ab,ti OR azunaftil:ab,ti OR praxilene:ab,ti OR nafronyloxalate:ab,ti 'OR oxalate, nafronyl':ab,ti | 1569 |
| #14 | propentofylline | 'propentofylline'/exp OR propentofylline:ab,ti | 686 |
| #15 | cilostazol | 'cilostazol'/exp OR cilostazol:ab,ti OR pletal:ab,ti | 6261 |
| #16 | Nicergoline | 'nicergoline'/exp OR nicergoline:ab,ti  OR nimergoline:ab,ti OR nicotergoline:ab,ti OR ergobe:ab,ti OR nicergobeta:ab,ti OR sermion:ab,ti  OR neuraxpharm:ab,ti OR nicerium:ab,ti | 1418 |
| #17 | Ginkgo biloba | 'ginkgo biloba'/exp OR 'ginkgo biloba' OR (('ginkgo'/exp OR ginkgo) AND biloba) OR 'ginkgo biloba':ab,ti OR 'ginkgo bilobas':ab,ti OR 'bilobas, ginkgo':ab,ti OR gingko:ab,ti OR gingkos:ab,ti | 11048 |
| #18 | Vitamin E | 'vitamin e'/exp OR 'vitamin e' OR (('vitamin'/exp OR vitamin) AND e) OR 'vitamin e':ab,ti | 269116 |
| #19 | Vitamins B | 'vitamins b' OR (('vitamins'/exp OR vitamins) AND b) OR 'vitamins b':ab,ti | 727534 |
| #20 | almitrine | 'almitrine'/exp OR almitrine:ab,ti  OR 'almitrine monomesylate':ab,ti OR vectarion:ab,ti OR 'almitrine dimesylate':ab,ti OR 'almitrine bis':ab,ti 'OR almitrine bismesylate':ab,ti | 1234 |
| #21 |  | #3 OR #4 OR #5 OR #6 OR #7 OR #8 OR #9 OR #10#11 OR #12 OR #13 OR #14 OR #15 OR #16 OR #17 OR #18#19 OR #20 | 1047631 |
| #22 | Final query | #1 AND #2 AND #21 | 2717 |

##

## Search strategies for Web of Science

| Step | Topic or intervention | Search algorithm | Items found |
| --- | --- | --- | --- |
| #1 | Disease | TS= Alzheimer disease | 167918 |
| #2 | Study design | TS=Randomized controlled trial | 443039 |
| #3 | Donepezil | TS=donepezil OR  TS=donepezil hydrochloride OR  TS=donepezilium oxalate trihydrate | 6204 |
| #4 | Rivastigmine | TS= rivastigmine OR  TS= rivastigmine tartrate OR  TS= rivastigmine hydrogen tartrate OR  TS= hydrogen tartrate, rivastigmine OR  TS= tartrate, rivastigmine hydrogen OR  TS= exelon | 2812 |
| #5 | Galantamine | TS= galantamine OR  TS= lycoremine OR  TS= galantamin OR  TS= nivaline OR  TS= nivalin OR  TS= razadyne OR  TS= reminyl OR  TS= galanthamine hydrobromide | 2666 |
| #6 | huperzine | TS= Huperzine OR  TS= huperzine a | 1227 |
| #7 | Memantine | TS= memantine OR  TS= Namenda OR  TS= ebixa OR  TS= memantine hydrochloride OR  TS= axura | 5169 |
| #8 | nimodipine | TS= nimodipine OR  TS= brainal OR  TS =calnit OR  TS= kenesil OR  TS= nymalize OR  TS= nimodipin hexal OR  TS= nimodipin isis OR  TS= nimodipino bayvit OR  TS= nimotop OR  TS= remontal OR  TS= admon modus | 4410 |
| #9 | aspirin | TS= nimodipine OR  TS= brainal OR  TS =calnit OR  TS= kenesil OR  TS= nymalize OR  TS= nimodipin hexal OR  TS= nimodipin isis OR  TS= nimodipino bayvit OR  TS= nimotop OR  TS= remontal OR  TS= admon modus | 4410 |
| #10 | aspirin | TS= aspirin OR  TS= acetylsalicylic Acid OR  TS = acid, acetylsalicylic OR  TS= 2-(acetyloxy)benzoic acid OR  TS= acylpyrin OR  TS= acetysal OR  TS= solupsan OR  TS= polopiryna OR | 65740 |
| #11 | citicoline | TS= citicoline OR  TS= choline, cytidine diphosphate OR  TS = diphosphate choline, cytidine OR  TS= cidifos OR  TS= citicoline OR  TS= cidifos OR  TS= citicoline OR  TS= choline, cdp OR  TS= cytidine 5-diphosphocholine | 1441 |
| #12 | **vinpocetine** | TS= vinpocetine OR  TS= kavinton OR  TS = cavinton | 717 |
| #13 | sertraline | TS= sertraline OR  TS= ertraline OR  TS = zoloft OR  TS= altruline OR  TS= apo sertraline OR  TS= besitran | 7090 |
| #14 | **Nafronyl** | TS= nafronyl OR  TS= naftidrofuryl OR  TS = azunaftil OR  TS= praxilene OR  TS= nafronyloxalate OR  TS= oxalate, nafronyl | 346 |
| #15 | propentofylline | TS= propentofylline | 361 |
| #16 | cilostazol | TS= cilostazol OR  TS= pletal | 2348 |
| #17 | Nicergoline | TS= nicergoline OR  TS= nimergoline OR  TS = nicotergoline OR  TS= ergobe OR  TS= nicergobeta OR  TS= sermion OR  TS= neuraxpharm OR  TS= nicerium | 245 |
| #18 | Ginkgo biloba | TS= ginkgo biloba OR  TS= ginkgo bilobas OR  TS = bilobas, ginkgo OR  TS= gingko OR  TS= gingkos | 6535 |
| #19 | Vitamin E | TS= vitamin e | 65212 |
| #20 | Vitamins B | TS='vitamins b' | 43643 |
| #21 | almitrine | TS= almitrine OR  TS= almitrine monomesylate OR  TS = vectarion OR  TS= almitrine dimesylate OR  TS= almitrine bis OR  TS= almitrine bismesylate | 406 |
| #23 |  | #3 OR #4 OR #5 OR #6 OR #7 OR #8 OR #9 OR #10#11 OR #12 OR #13 OR #14 OR #15 OR #16 OR #17 OR #18#19 OR #20 OR #21 OR #22 | 199648 |
| #24 | Final query | #23 AND #2 AND #1 | 1085 |

##

## Search strategies for Cochrane Central Register of Controlled Trials

| Step | Topic or intervention | Search algorithm | Items found |
| --- | --- | --- | --- |
| #1 | Disease | MeSH descriptor: [Alzheimer Disease] explode all trees OR  (Alzheimer Disease): ti,ab,kw OR  (Alzheimer Syndrome): ti,ab,kw OR  (Alzheimer's Diseases): ti,ab,kw | 11072 |
| #2 | Study design | MeSH descriptor: [Randomized OR Controlled Trial] explode all trees OR  MeSH descriptor: [Randomized Controlled Trial] explode all trees OR  randomised controlled trial:ti,ab,kw OR randomised controlled trial:ti,ab,kw | 209346 |
| #3 | Donepezil | MeSH descriptor: [Donepezil] explode all trees OR (Donepezil): ti,ab,kw OR  Donepezil Hydrochloride:ti,ab,kw OR  Aricept:ti,ab,kw | 1726 |
| #4 | Rivastigmine | MeSH descriptor: [Rivastigmine] explode all trees OR (Rivastigmine):ti,ab,kw OR  Rivastigmine Tartrate:ti,ab,kw OR  Rivastigmine Hydrogen Tartrate:ti,ab,kw OR Hydrogen Tartrate, Rivastigmine: ti,ab,kw OR(Hydrogen Tartrate, Rivastigmine):ti,ab,kw OR Exelon:ti,ab,kw | 136151 |
| #5 | Galantamine | (Galantamine):ti,ab,kw OR  (Lycoremine):ti,ab,kw OR  (Galantamin):ti,ab,kw OR  (Nivaline):ti,ab,kw OR  (Nivalin):ti,ab,kw OR  (Razadyne) :ti,ab,kw OR  (Reminyl):ti,ab,kw OR  (alanthamine Hydrobromide) :ti,ab,kw | 72 |
| #6 | huperzine | (huperzine):ti,ab,kw  (huperzine A):ti,ab,kw | 74 |
| #7 | Memantine | MeSH descriptor: [Memantine] explode all trees OR (Memantine):ti,ab,kw OR  (Memantine):ti,ab,kw OR  (Ebixa):ti,ab,kw OR  (Axura):ti,ab,kw | 1286 |
| #8 | nimodipine | MeSH descriptor: [Nimodipine] explode all trees OR  (nimodipine):ti,ab,kw OR  (Brainal):ti,ab,kw OR  (Calnit):ti,ab,kw OR  (Nymalize):ti,ab,kw OR  (Nimodipin Hexal):ti,ab,kw OR  (Nimodipin-ISIS):ti,ab,kw OR  (Nimotop):ti,ab,kw | 812 |
| #9 | aspirin | MeSH descriptor: [Aspirin] explode all trees OR(Aspirin):ti,ab,kw OR  (Acetylsalicylic Acid):ti,ab,kw OR  (Acid, Acetylsalicylic):ti,ab,kw OR  (Acylpyrin):ti,ab,kw OR  (Aloxiprimum):ti,ab,kw OR  (Acetysal):ti,ab,kw OR  (Solupsan):ti,ab,kw OR  (Polopiryna):ti,ab,kw | 16868 |
| #10 | citicoline | MeSH descriptor: [Cytidine Diphosphate Choline] explode all trees OR  (citicoline):ti,ab,kw OR  Choline, Cytidine Diphosphate:ti,ab,kw OR Diphosphate Choline, Cytidine:ti,ab,kw OR  Cidifos:ti,ab,kw OR  Citicoline:ti,ab,kw OR  Choline, CDP:ti,ab,kw | 372 |
| #11 | **vinpocetine** | (vinpocetine):ti,ab,kw OR  ethyl apovincaminate:ti,ab,kw OR  Kavinton:ti,ab,kw OR  Cavinton:ti,ab,kw OR | 119 |
| #12 | sertraline | MeSH descriptor: [Sertraline] explode all trees OR sertraline:ti,ab,kw OR  Zoloft:ti,ab,kw OR  Altruline:ti,ab,kw OR  Apo-Sertraline:ti,ab,kw OR  Besitran:ti,ab,kw OR  Sertraline Hydrochloride:ti,ab,kw OR  Hydrochloride, Sertraline:ti,ab,kw OR  Rhoxal sertraline:ti,ab,kw | 2642 |
| #13 | **Nafronyl** | MeSH descriptor: [Nafronyl] explode all trees OR (Nafronyl) :ti,ab,kw OR  Naftidrofury:ti,ab,kw OR  Azunaftil: ti,ab,kw OR  Praxilene: ti,ab,kw OR  Nafronyloxalate :ti,ab,kw OR  Oxalate, Nafronyl: ti,ab,kw | 106 |
| #14 | propentofylline | (propentofylline): ti,ab,kw | 57 |
| #15 | cilostazol | MeSH descriptor: [cilostazol] explode all trees OR (cilostazol): ti,ab,kw OR  Pletal:ti,ab,kw | 846 |
| #16 | Nicergoline | MeSH descriptor: [Nicergoline] explode all trees OR  (Nicergoline) :ti,ab,kw OR  Nimergoline :ti,ab,kw OR  Nicotergoline:ti,ab,kw OR  Ergobel:ti,ab,kw OR  Nicergobeta:ti,ab,kw OR  Ergobel:ti,ab,kw OR  Sermion:ti,ab,kw OR  Nicergolin Lindo:ti,ab,kw OR  Nicergolin-Neuraxpharm :ti,ab,kw OR  Nicergolin Atid:ti,ab,kw OR  Nicergolin TEVA :ti,ab,kw OR | 137 |
| #17 | Ginkgo biloba | MeSH descriptor: [Ginkgo biloba] explode all trees OR  (Ginkgo biloba):ti,ab,kw OR  Ginkgo bilobas:ti,ab,kw OR  bilobas, Ginkgo:ti,ab,kw OR  Gingko:ti,ab,kw OR  Gingkos:ti,ab,kw | 1137 |
| #18 | Vitamin E | MeSH descriptor: [Vitamin E] explode all trees OR Vitamin E:ti,ab,kw | 7656 |
| #19 | Vitamins B | (Vitamins B):ti,ab,kw | 5714 |
| #20 | almitrine | MeSH descriptor: [Almitrine] explode all trees OR  almitrine:ti,ab,kw OR  Almitrine Monomesylate:ti,ab,kw OR  Almitrine ,Monomesylate:ti,ab,kw OR  Vectarion:ti,ab,kw OR  Almitrine Dimesylate:ti,ab,kw OR  Almitrine Bis:ti,ab,kw OR  Almitrine Bismesylate:ti,ab,kw | 218 |
| #21 |  | #3 OR #4 OR #5 OR #6 OR #7 OR #8 OR #9 OR #10#11 OR #12 OR #13 OR #14 OR #15 OR #16 OR #17 OR #18#19 OR #20 | 169691 |
| #22 | Final query | #21 AND #2 AND #1 | 314 |

##

## Search strategies for Scopus

| Step | Topic or intervention | Search algorithm | Items found |
| --- | --- | --- | --- |
| #1 | Disease | TITLE-ABS-KEY (“Alzheimer disease")  OR TITLE-ABS-KEY ("Alzheimer Dementia")  OR TITLE-ABS-KEY ("Alzheimer Dementias")  OR TITLE-ABS-KEY ("Dementia, Alzheimer")  OR TITLE-ABS-KEY ("Alzheimer's Disease" )  OR TITLE-ABS-KEY ("Dementia, Senile" )  OR TITLE-ABS-KEY ("Senile Dementia" )  OR TITLE-ABS-KEY ("Dementia, Alzheimer Type" )  OR TITLE-ABS-KEY ("Alzheimer Type Dementia")  OR TITLE-ABS-KEY ("Alzheimer Type Senile Dementia")  OR TITLE-ABS-KEY ("Primary Senile Degenerative Dementia")  OR TITLE-ABS-KEY ("Dementia, Primary Senile Degenerative")  OR TITLE-ABS-KEY ("Alzheimer Sclerosis" )  OR TITLE-ABS-KEY  "Alzheimer Syndrome" )  OR TITLE-ABS-KEY ("Alzheimer's Diseases" )   OR TITLE-ABS-KEY ("Alzheimer Diseases") ) | 228023 |
| #2 | Study design | (TITLE-ABS KEY ("Randomized")  OR TITLE-ABS-KEY ("Randomised")  OR TITLE-ABS-KEY （"Randomization")  OR TITLE-ABS-KEY ("Randomisation" ) | 1,229,674 |
| #3 | Donepezil | (TITLE-ABS-KEY ( "Donepezil" )  OR TITLE-ABS-KEY ("donepezilium Oxalate Trihydrate" )  OR TITLE-ABS-KEY ("Eranz" )  OR TITLE-ABS-KEY ("donepezil Hydrochloride" )  OR TITLE-ABS-KEY ("Aricept" ) ) | **12,828** |
| #4 | Rivastigmine | (TITLE-ABS-KEY ( "Rivastigmine" )  OR TITLE-ABS-KEY ("Rivastigmine Hydrogen Tartrate" )  OR TITLE-ABS-KEY ("Hydrogen Tartrate, Rivastigmine" )  OR TITLE-ABS-KEY ("Tartrate, Rivastigmine Hydrogen" )  OR TITLE-ABS-KEY ("Exelon" ) ) | **7,461** |
| #5 | Galantamine | **(TITLE-ABS-KEY** **(** **"Galantamine"** **)****OR** **TITLE-ABS-KEY** **("Lycoremine"** **)****OR** **TITLE-ABS-KEY** （**"Galantamin"** **)****OR** **TITLE-ABS-KEY** **("Galantamin"** **)****OR** **TITLE-ABS-KEY** **("Nivalin"** **)****OR** **TITLE-ABS-KEY** **("Razadyne"** **)****OR** **TITLE-ABS-KEY** **("Reminyl"** **)****OR** **TITLE-ABS-KEY** **("Galanthamine Hydrobromide"** **)** **)** | **7,692** |
| #6 | huperzine | **(TITLE-ABS-KEY** **(** **"huperzine"** **)****OR** **TITLE-ABS-KEY** **("huperzine a"** **)** **)** | **1,618** |
| #7 | Memantine | **(TITLE-ABS-KEY** **(** **"Memantine"** **)****OR** **TITLE-ABS-KEY** **("Namenda"** **)****OR** **TITLE-ABS-KEY** **("Ebixa"** **)****OR** **TITLE-ABS-KEY** **("Memantine Hydrochloride"** **)****OR** **TITLE-ABS-KEY** **("Axura"** **)** **)** | **10,224** |
| #8 | nimodipine | **(TITLE-ABS-KEY** **(** **"nimodipine"** **)****OR** **TITLE-ABS-KEY** **("Brainal"** **)****OR** **TITLE-ABS-KEY** **("Calnit"** **)****OR** **TITLE-ABS-KEY** **("Kenesil"** **)****OR** **TITLE-ABS-KEY** **("Nymalize"** **)****OR** **TITLE-ABS-KEY** **("Nimodipin Hexal"** **)****OR** **TITLE-ABS-KEY** **("Nimodipin Hexal"** **)****OR** **TITLE-ABS-KEY** **("Nimodipin-ISIS"** **)****OR** **TITLE-ABS-KEY** **("Nimodipino Bayvit"** **)****OR** **TITLE-ABS-KEY** **("Nimotop "** **)****OR** **TITLE-ABS-KEY** **("Remontal"** **)****OR** **TITLE-ABS-KEY** **("Admon Modus "** **)** | **10,757** |
| #9 | aspirin | **(TITLE-ABS-KEY** **(** **"Aspirin"** **)****OR** **TITLE-ABS-KEY** **("Acetylsalicylic Aci"** **)****OR** **TITLE-ABS-KEY** **("Acetylsalicylic Aci"** **)****OR** **TITLE-ABS-KEY** **("2-(Acetyloxy)benzoic Acid"** **)****OR** **TITLE-ABS-KEY** **("Acylpyrin"** **)****OR** **TITLE-ABS-KEY** **("Aloxiprimum"** **)****OR** **TITLE-ABS-KEY** **(** **"Acetysal"** **)****OR** **TITLE-ABS-KEY** **(** **"Solupsan"** **)****OR** **TITLE-ABS-KEY** **(** **"Polopiryna"** **)** **)** | **216,968** |
| #10 | citicoline | **(** **TITLE-ABS-KEY** **(** **"citicoline"** **)****OR** **TITLE-ABS-KEY** **(** **"Choline,Cytidine Diphosphate"** **)****OR** **TITLE-ABS-KEY** **(** **"Choline,Cytidine Diphosphate"** **)****OR** **TITLE-ABS-KEY** **(** **"Cidifos"** **)****OR** **TITLE-ABS-KEY** **(** **"Citicoline"** **)****OR** **TITLE-ABS-KEY** **(** **"Citicoline"** **)****OR** **TITLE-ABS-KEY** **(** **"Choline, CDP"** **)****OR** **TITLE-ABS-KEY** **(** **"Cytidine5'-Diphosphocholine"** **)** **)** | **2,142** |
| #11 | **vinpocetine** | **(** **TITLE-ABS-KEY** **(** **"vinpocetine"** **)****OR** **TITLE-ABS-KEY** **(** **"ethyl apovincaminate"** **)****OR** **TITLE-ABS-KEY** **(** **"Kavinton"** **)****OR** **TITLE-ABS-KEY** **(** **"Cavinton"** **)** **)** | **1,577** |
| #12 | sertraline | **TITLE-ABS-KEY** **(** **"sertraline"** **)****OR** **TITLE-ABS-KEY** **(** **"Zoloft"** **)****OR** **TITLE-ABS-KEY** **(** **"Altruline Sertraline "** **)****OR** **TITLE-ABS-KEY** **(** **"Besitran"** **)****OR** **TITLE-ABS-KEY** **(** **"Sertraline Hydrochloride"** **)****OR** **TITLE-ABS-KEY** **(** **"Hydrochloride,** **Sertraline"** **)****OR** **TITLE-ABS-KEY** **(** **"Rhoxal sertraline"** **)** **)** | **26,092** |
| #13 | **Nafronyl** | **(** **TITLE-ABS-KEY** **(** **"Nafronyl"** **)****OR** **TITLE-ABS-KEY** **(** **"Naftidrofuryl"** **)****OR** **TITLE-ABS-KEY** **(** **"Azunaftil "** **)****OR** **TITLE-ABS-KEY** **(** **"Praxilene"** **)****OR** **TITLE-ABS-KEY** **(** **"Praxilene"** **)****OR** **TITLE-ABS-KEY** **(** **"Oxalate, Nafronyl"** **)****OR** **TITLE-ABS-KEY** **(** **"Nafronyloxalate"** **)** **)** | **1,750** |
| #14 | propentofylline | **TITLE-ABS-KEY** **(** **"propentofylline"** **)** | **656** |
| #15 | cilostazol | **(TITLE-ABS-KEY** **(** **"cilostazol"** **)****OR** **TITLE-ABS-KEY** **(** **"Pletal"** **)** **)** | **5,403** |
| #16 | Nicergoline | **(** **TITLE-ABS-KEY** **(** **"Nicergoline"** **)****OR** **TITLE-ABS-KEY** **(** **"Nimergoline"** **)****OR** **TITLE-ABS-KEY** **(** **"Nicotergoline"** **)****OR** **TITLE-ABS-KEY** **(** **"Ergobel"** **)****OR** **TITLE-ABS-KEY** **(** **"Nicergobeta"** **)****OR** **TITLE-ABS-KEY** **(** **"Sermion"** **)****OR** **TITLE-ABS-KEY** **(** **"Nicergolin Lindo"** **)****OR** **TITLE-ABS-KEY** **(** **"Nicergolin-Neuraxpharm "** **)****OR** **TITLE-ABS-KEY** **(** **"Nicerium"** **)****OR** **TITLE-ABS-KEY** **(** **"Nicergolin Atid"** **)****OR** **TITLE-ABS-KEY** **(** **"Nicergolin TEVA"** **)** **)** | **1,403** |
| #17 | Ginkgo biloba | **(** **TITLE-ABS-KEY** **(** **"Ginkgo biloba"** **)****OR** **TITLE-ABS-KEY** **(** **"Ginkgo bilobas"** **)****OR** **TITLE-ABS-KEY** **(** **"bilobas, Ginkgo"** **)****OR** **TITLE-ABS-KEY** **(** **"Ginkgo"** **)****OR** **TITLE-ABS-KEY** **(** **"Gingkos "** **)** **)** | **12,600** |
| #18 | Vitamin E | **TITLE-ABS-KEY** **(** **"Vitamin E"** **)** | **51,014** |
| #19 | Vitamins B | **TITLE-ABS-KEY** **(** **"Vitamins B"** **)** | **45,990** |
| #20 | almitrine | （ **TITLE-ABS-KEY** **(** **"almitrine"** **)****OR** **TITLE-ABS-KEY** **(** **"Almitrine Monomesylate"** **)****OR** **TITLE-ABS-KEY** **(** **"Almitrine ,Monomesylate"** **)****OR** **TITLE-ABS-KEY** **(** **"Vectarion"** **)****OR** **TITLE-ABS-KEY** **(** **"Almitrine Dimesylate"** **)****OR** **TITLE-ABS-KEY** **(** **"Almitrine Bis"** **)****OR** **TITLE-ABS-KEY** **(** **"Almitrine Bismesylate"** **)** **)** | **1,222** |
| #21 |  | #3 OR #4 OR #5 OR #6 OR #7 OR #8 OR #9 OR #10#11 OR #12 OR #13 OR #14 OR #15 OR #16 OR #17 OR #18#19 OR #20 | 281603 |
| #22 | Final query | #21 AND #2 AND #1 | 2116 |

## Search strategies for Embase

| Step | Topic or intervention | Search algorithm | Items found |
| --- | --- | --- | --- |
| #1 | Disease | 'alzheimer disease'/exp OR 'alzheimer disease' OR (alzheimer AND ('disease'/exp OR disease)) OR 'alzheimer disease':ti,ab,kw OR'alzheimer disease':ti,ab,kw OR 'alzheimer dement*':ti,ab,kw OR'alzheimers dise*':ti,ab,kw OR 'alzheimer syndr*':ti,ab,kw | 278496 |
| #2 | Study design | 'randomized controlled trial'/exp OR 'randomized controlled trial':ti,ab,it OR 'randomized':ti,ab,it OR 'randomised':ti,ab,it OR 'randomization':ti,ab,it OR 'randomisation':ti,ab,it OR rct:ti,ab,it | 1192617 |
| #3 | Donepezil | 'donepezil'/exp OR 'donepezil' ab,ti OR  'donepezilium oxalate trihydrate':ab,ti  OR eranz:ab,ti OR 'donepezil hydrochloride':ab,ti OR aricept:ab,ti | 14092 |
| #4 | Rivastigmine | 'rivastigmine'/exp OR 'rivastigmine tartrate': ab,ti OR 'rivastigmine hydrogen tartrate':ab,ti OR 'hydrogen tartrate, rivastigmine':ab,ti OR 'tartrate, rivastigmine hydrogen':ab,ti OR exelon:ab,ti | 7937 |
| #5 | Galantamine | 'galantamine'/exp OR galantamine:ab,ti  OR lycoremine:ab,ti OR galantamin:ab,ti  OR nivaline:ab,ti OR nivalin:ab,ti OR  razadyne:ab,ti OR reminyl:ab,ti OR 'galanthamine hydrobromide':ab,ti | 8169 |
| #6 | huperzine | Huperzine /exp OR huperzine:ab,ti OR 'huperzine a':ab,ti | 1581 |
| #7 | Memantine | 'memantine'/exp OR memantine:ab,ti OR namenda:ab,ti OR ebixa:ab,ti  OR 'memantine hydrochloride':ab,ti OR axura:ab,ti | 11659 |
| #8 | nimodipine | 'nimodipine'/exp OR nimodipine:ab,ti  OR brainal:ab,ti OR calnit:ab,ti OR kenesil:ab,ti OR nymalize:ab,ti OR 'nimodipin hexal':ab,ti OR 'nimodipin isis':ab,ti OR 'nimodipino bayvit':ab,ti OR nimotop:ab,ti OR remontal:ab,ti  OR 'admon modus':ab,ti | 11083 |
| #9 | aspirin | ‘aspirin’/exp OR aspirin:ab,ti OR ‘acetylsalicylic Acid’ :ab,ti OR ‘acid, acetylsalicylic’ :ab,ti OR ‘2-(acetyloxy)benzoic acid’ :ab,ti OR acylpyrin :ab,ti OR aloxiprimum :ab,ti OR acetysal :ab,ti OR solupsan:ab,ti OR polopiryna:ab,ti | 23544 |
| #10 | citicoline | 'citicoline'/exp OR citicoline:ab,ti OR 'choline, cytidine diphosphate':ab,ti  OR 'diphosphate choline, cytidine':ab,  OR cidifos:ab,ti OR citicoline:ab,ti  OR 'choline, cdp':ab,ti OR 'cytidine 5-diphosphocholine':ab,ti | 2367 |
| #11 | **vinpocetine** | 'vinpocetine'/exp OR vinpocetine:ab,ti  OR vinpocetine:ab,ti OR kavinton:ab,ti  OR cavinton:ab,ti | 1634 |
| #12 | sertraline | sertraline /exp OR sertraline:ab,ti OR ertraline:ab,ti OR zoloft:ab,ti  OR altruline:ab,ti OR 'apo sertraline':ab,ti OR besitran:ab,ti | 346 |
| #13 | **Nafronyl** | 'nafronyl'/exp OR nafronyl:ab,ti OR naftidrofuryl:ab,ti OR azunaftil:ab,ti  OR praxilene:ab,ti OR nafronyloxalate:ab,ti 'OR oxalate, nafronyl':ab,ti | 1569 |
| #14 | propentofylline | 'propentofylline'/exp OR propentofylline:ab,ti | 686 |
| #15 | cilostazol | 'cilostazol'/exp OR cilostazol:ab,ti OR pletal:ab,ti | 6261 |
| #16 | Nicergoline | 'nicergoline'/exp OR nicergoline:ab,ti  OR nimergoline:ab,ti OR nicotergoline:ab,ti OR ergobe:ab,ti OR nicergobeta:ab,ti OR sermion:ab,ti OR neuraxpharm:ab,ti OR nicerium:ab,ti | 1418 |
| #17 | Ginkgo biloba | 'ginkgo biloba'/exp OR 'ginkgo biloba' OR (('ginkgo'/exp OR ginkgo) AND biloba) OR 'ginkgo biloba':ab,ti OR 'ginkgo bilobas':ab,ti OR 'bilobas, ginkgo':ab,ti OR gingko:ab,ti OR gingkos:ab,ti | 11048 |
| #18 | Vitamin E | 'vitamin e'/exp OR 'vitamin e' OR (('vitamin'/exp OR vitamin) AND e) OR 'vitamin e':ab,ti | 269116 |
| #19 | Vitamins B | 'vitamins b' OR (('vitamins'/exp OR vitamins) AND b) OR 'vitamins b':ab,ti | 727534 |
| #20 | almitrine | 'almitrine'/exp OR almitrine:ab,ti  OR 'almitrine monomesylate':ab,ti OR vectarion:ab,ti OR 'almitrine dimesylate':ab,ti OR 'almitrine bis':ab,ti 'OR almitrine bismesylate':ab,ti | 1234 |
| #21 |  | #3 OR #4 OR #5 OR #6 OR #7 OR #8 OR #9 OR #10#11 OR #12 OR #13 OR #14 OR #15 OR #16 OR #17 OR #18#19 OR #20 | 1047631 |
| #22 | Final query | #21 AND #2 AND #1 | 2717 |
