# Supplementary material for: Effect of long-term pharmacological treatments on Alzheimer disease: A systematic review and network meta-analysis
Source: Medicine (Baltimore). 2024 Sep 20;103(38):e39753. doi: 10.1097/MD.0000000000039753 (PMC11419515; doi:10.1097/MD.0000000000039753)

Supplementary Figure 1: Risk of bias graph

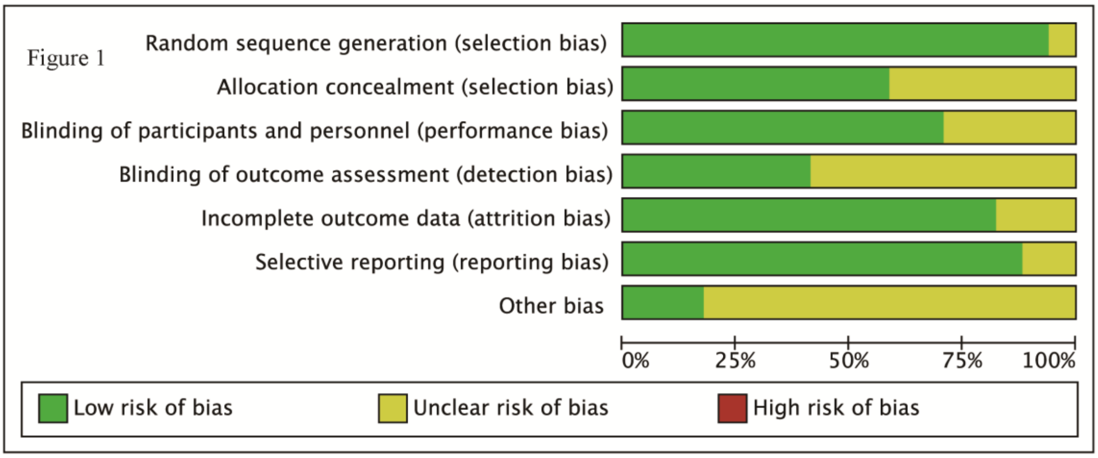

Supplementary Figure 2: Risk of bias summary

Figure 2

	Random sequence generation (selection bias)	Allocation concealment (selection bias)	Blinding of participants and personnel (performance bias)	Blinding of outcome assessment (detection bias)	Incomplete outcome data (attrition bias)	Selective reporting (reporting bias)	Other bias
Aisen PS2008	+	+	+	+	+	+	?
Baoli Z 2013	+	+	+	?	?	+	+
Bullock R 2005	+	?	+	?	+	+	?
Bullock R 2006	+	?	+	?	+	+	?
Farlow M 2000	?	?	?	+	+	+	+
Hager K 2014	+	+	+	+	+	+	?
Haih Huang 2011	+	+	?	?	+	+	?
Haihua H 2011	+	+	?	?	?	+	+
Karaman Y 2005	+	?	+	?	+	+	?
Le Bar PL 1997	+	+	+	+	+	+	?
Le Bars PL 2002	+	?	+	?	+	+	?
Mohs RC 2001	+	+	+	+	+	+	?
Peters O 2015	+	?	+	?	+	?	?
Sparks DL 2005	+	+	+	+	+	?	?
Wilkinson D 2012	+	+	?	?	+	+	?
Winblad B 2001	+	?	?	?	?	+	?
Yokoyama S 2019	+	+	+	+	+	+	?

Supplementary Figure 3: Assessment of the inconsistency results for cognition; A: Placebo; B: Rivastigmine 12 mg; C: Rivastigmine 4 mg

Figure 3

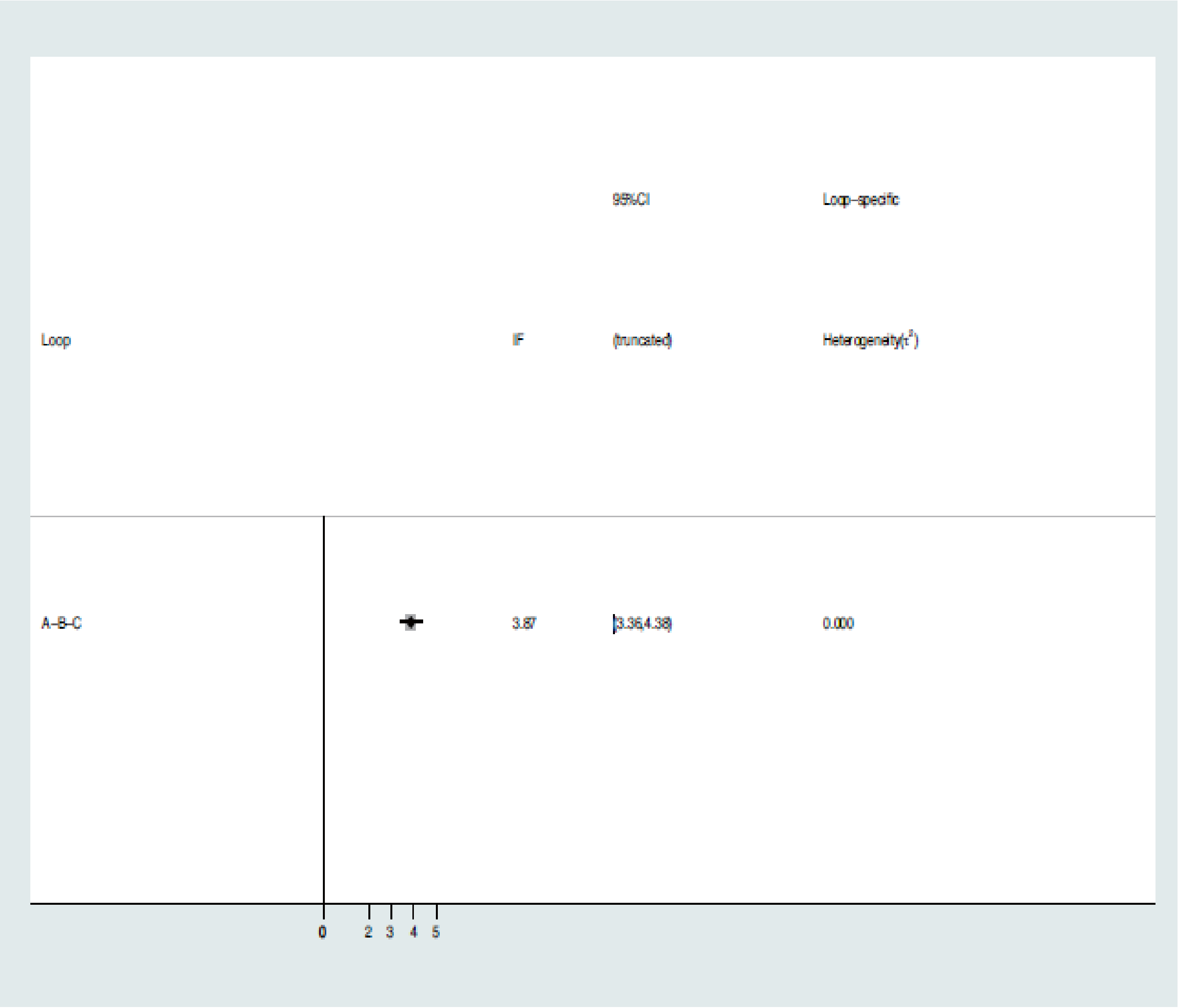

Supplementary Figure 4 : Funnel plot for cognition from the network meta-analysis; A: Placebo; B: Rivastigmine 12 mg; C: Rivastigmine 4 mg; D: EGb120 mg; E: Memantine 20 mg; F:Rivastigmine12 mg+ EGb120 mg; G:Donepezil10mg;H:VitaminB; I: Atorvastatin-calcium 80 mg;

Figure 4

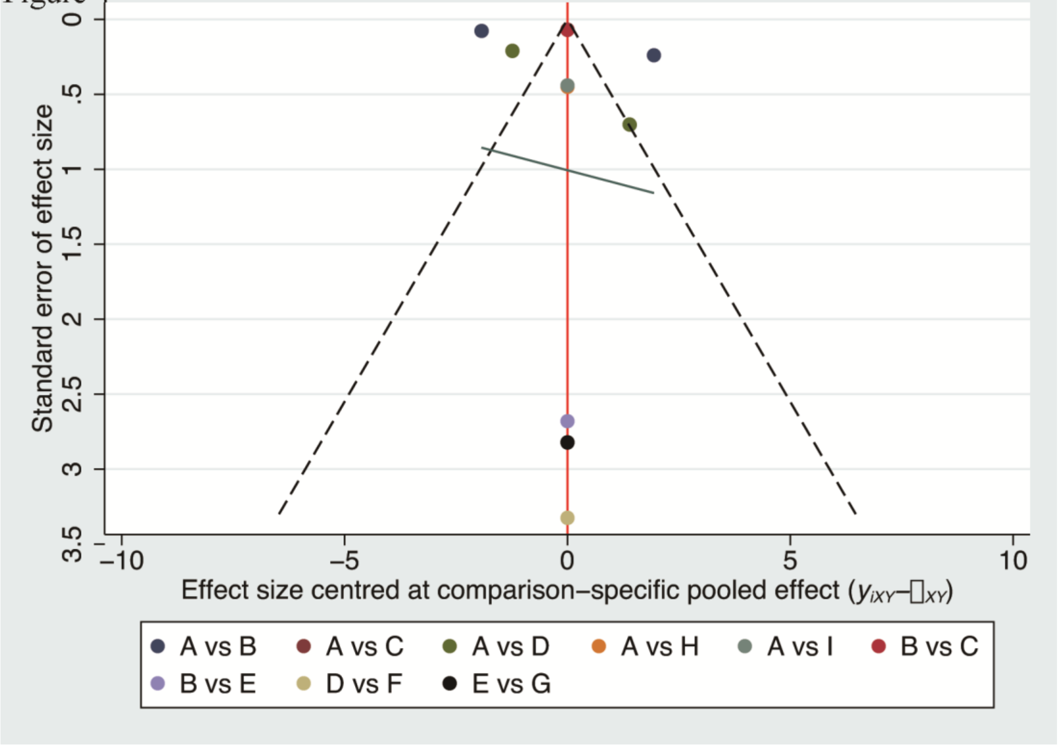

Supplementary Figure 5: Assessment of the inconsistency results for safety

Figure 5

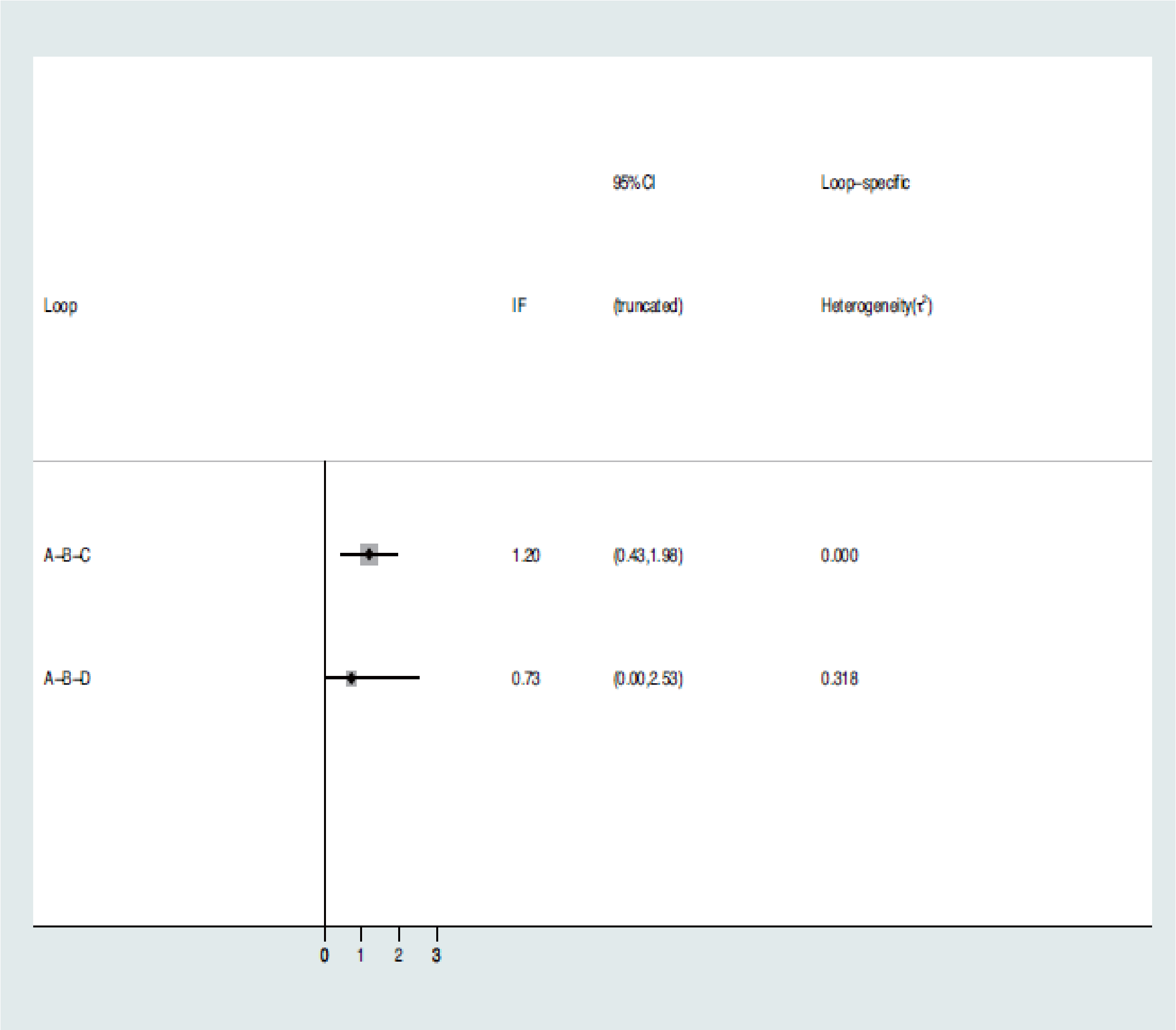

Supplementary Figure 6: Funnel plot for safety from the network meta-analysis; A: Placebo; B: Rivastigmine 12 mg; C: Rivastigmine 4mg;D:Donepezil10mg;E:Memantine 20 mg; F: Galantamine 24 mg; I: Memantine 20 mg +Galantamine 24 mg

Figure 6

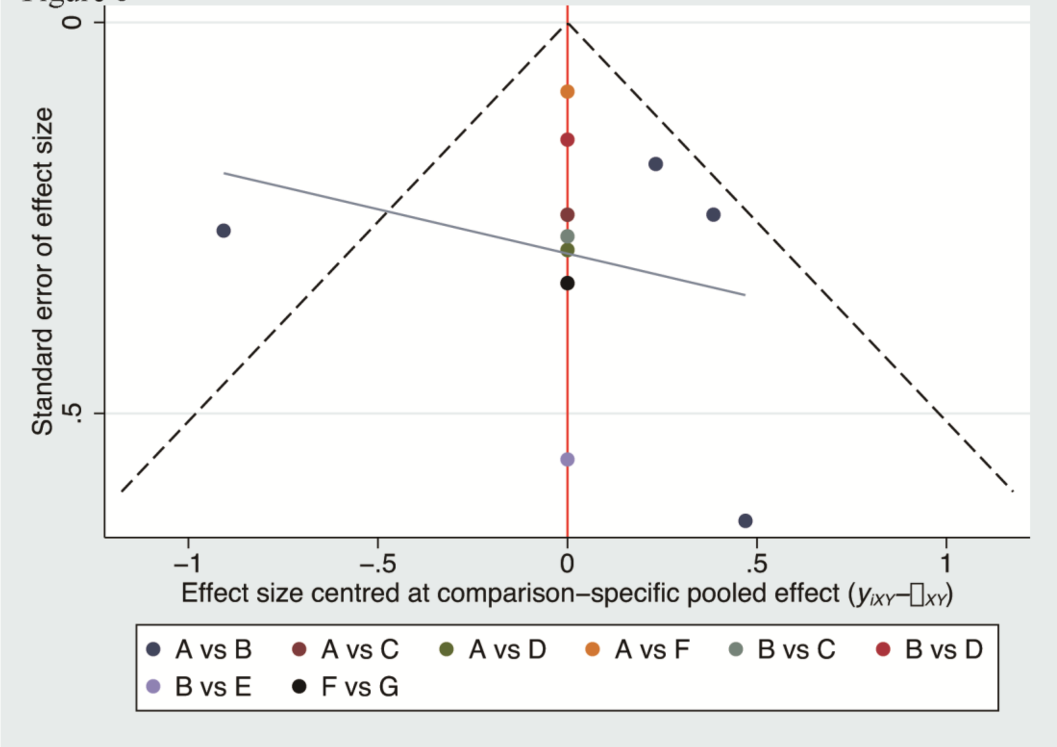

Supplement: Supplementary file 2 [file medi-103-e39753-s002.pdf]
